# *Bacillus* and biopolymer: Prospects and challenges

**DOI:** 10.1016/j.bbrep.2017.10.001

**Published:** 2017-10-21

**Authors:** Swati Mohapatra, Sudipta Maity, Hirak Ranjan Dash, Surajit Das, Swati Pattnaik, Chandi Charan Rath, Deviprasad Samantaray

**Affiliations:** a Department of Biotechnology, Indian Institute of Technology, Roorkee 247667, India; b Department of Microbiology, CPGS, OUAT, Bhubaneswar-3, Odisha, India; c Department of Life Science, National Institute of Technology, Rourkela 769008, Odisha, India; d Department of Botany, CBSH, OUAT, Bhubaneswar-3, Odisha, India

**Keywords:** PHAs, *Bacillus*, Biopolymer, Biodegradability, Biogenesis

## Abstract

The microbially derived polyhydroxyalkanoates biopolymers could impact the global climate scenario by replacing the conventional non-degradable, petrochemical-based polymer. The biogenesis, characterization and properties of PHAs by *Bacillus* species using renewable substrates have been elaborated by many for their wide applications. On the other hand *Bacillus* species are advantageous over other bacteria due to their abundance even in extreme ecological conditions, higher growth rates even on cheap substrates, higher PHAs production ability, and the ease of extracting the PHAs. *Bacillus* species possess hydrolytic enzymes that can be exploited for economical PHAs production. This review summarizes the recent trends in both non-growth and growth associated PHAs production by *Bacillus* species which may provide direction leading to future research towards this growing quest for biodegradable plastics, one more critical step ahead towards sustainable development.

## Highlights

• Biogenesis, characterization and properties of PHAs generated by *Bacillus* species.
• Strategies for economic PHAs production by *Bacillus* species and its applications.
• Prospects and challenge of using *Bacillus* species for PHAs production.

## 1 Introduction

In developing countries several activities are transforming local problems into international issues in this global village. Plastics with favourable mechanical integrity and excellent durability have been one of the fall-outs of the rapid progress in material science technology. Having its utility in diverse sectors, plastics have became an essential part of the modern life. In the global commodity petrochemical based plastic production has grown two hundred fold from 1.5 million tons in 1950 to 299 million tons with an annual growth rate of 9% in 2013 [1], [2]. These are typical petroleum-based, non-biodegradable polymers gather or aggregate around our ecosystem which is a far cry from few years back ecosystem [2]. Degradation of such solid wastes is a global concern. Even though it is difficult to completely ban the use of plastics due to their versatile utilities, it is possible to replace or reduce their use with alternative biodegradable polymers with similar properties.

Among the entire bio-based and bio-degradable polymer, polyhydroxyalkanoates (PHAs) are well-known. These are bio-based and biodegradable without waste and also recycled to CO_2_ and water. The endocellular PHAs are biosynthesized hydroxy-fatty-acids stored as lipid inclusions when carbon source is in abundance and nutrients like nitrogen, phosphorus, oxygen or sulphur are limited. These are secondary metabolites produced by various microbes in response to environmental stress. Such microorganisms can be located in diverse ecological niches like costal water body sediments, marine region, rhizospheric soil, water sediments and sludge [3]. These environments are often brimming over with organic nutrients and poor in other nutrients to support active growth and meet the metabolic requirements of the starving PHAs accumulating microbial population [4]. Extensive research provides a clear vision on several PHAs producers, that these microbes synthesize PHAs inclusions in the late log phase of growth cycle. Then, in later stage of their life cycle they use it as a carbonosoms [5], [6]. Through metabolic activities, PHA is normally depolymerized to D-hydroxy-butyrate on demand, and then metabolized to acetoacetate and acetoacetyl-CoA [7] to provide energy to the cell.

Though these carbonosoms accumulation has been investigated in various bacterial isolates, *Bacillus* species are extensively studied in PHAs world since the exploration of poly-*β*-hydroxybutyrate (PHB) in the cytosol of *Bacillus megaterium* by the French Lemoigne, in 1926 [8]. Some *Bacillus* species have been reported to produce as much as 90% (w/w) PHAs of dry cells during nutrients imbalance [9]. *Bacillus* species becoming model organisms in industry and academic world attributed primarily to its genetic stability [10]. Apart from higher growth rate compared to other bacteria, the use of *Bacillus* species to produce PHAs is advantageous over others due to the absence of lipopolysaccharides external layer in them which makes PHAs extraction much simple [11]. *Bacillus* species are also capable of producing PHAs copolymers utilizing the relatively simple, inexpensive and structurally unrelated carbon sources. Moreover, the isolates possess the ability to secrete number of hydrolytic enzymes that can be exploited for cost affordable PHAs production by utilizing, for instance, agro-industrial and other waste materials [12].

The major drawback of *Bacillus* species in PHAs production is their sporulating nature. Practically the fact of sporulation and deposition of PHAs granules provoked due to stress factors [13]. To overcome the predicament research on pilot scale PHB productions by *B. cereus* in the media that depresses sporulation, under acidic pH [14] and potassium deficiency [15] conditions. These pores over strategies not only inhibit spore formation in *Bacillus* but also can enhance the PHAs productivity. Several studies of PHAs are dealing with mostly on upstream and downstream process, its applications [16], [17] and with genetic modifications or mutations to increase the yield [9], [18]. Now these expertises become an impediment, being economically nonfeasible to market. This review summarizes these recent trends in PHAs production by *Bacillus* species as an effort to provide direction and leads to future research and development towards the growing quest for biodegradable plastics, one more critical step ahead towards an eco-sustainable development.

## 2 Biogenesis and chemistry

### 2.1 Diversity and synthesis of biopolymers by *Bacillus*

The genus *Bacillus* is capable of producing organic and inorganic intracellular spherical inclusion bodies enclosed by phospholipid-protein membrane in the cytosol. The inorganic inclusion bodies are magnetosomes surrounded by iron oxide core and the organic hydrophobic inclusion is PHAs surrounded by polyester core [19]. Evidently, the presence of PHAs granules in the microbial cytosol have also been served as a chemotaxonomic signature for detection of various isolates [20]. A wide array of PHAs producer *Bacillus* species (Table 1a) are recorded in the last few years with diverse biosynthetic mechanism, structural, thermal and functional properties.

**Table 1a:** PHAs produced from synthetic substrate by different species of *Bacillus*.

***Bacillus*****sp.**	**Substrate**	**PHAs yield (% of DCW)**	**Fermentation**	**PHAs type**	**Reference**
*Bacillus aryabhattai*	Sucrose, glucose & fructose	57.62	Batch	PHAs	[40]
*Bacillus cereus* SPV	Glucose	38.00	Batch	3HB & 3HV	[14]
*Bacillus cereus*	Glucose	13.77	–	PHB-3HHX	[34]
*Bacillus licheniformis*	Glucose	53.01	Batch	PHB	[42]
*Bacillus megaterium* uyuni S29	Glucose	70.00	Feed Batch	PHB	[37]
*Bacillus mycoides* DFC1	Glucose	57.20	Batch	PHB	[28]
*Bacillus mycoides* DFC1	Glucose	76.32	–	PHB	[35]
*Bacillus* sp.	Glucose	68.85	–	PHB	[26]
*Bacillus* sp.	Raffinose	60.57	Batch	P(3HB)	[27]
*Bacillus* sp.	Glucose	80	–	PHA	[57]
*Bacillus* sp.	Sucrose	51.49	Batch	PHAs	[41]
*Bacillus* sp. SW1-2	Glucose	36.00	Feed Batch	PHB	[36]
*Bacillus* sp. Ti3	Starch	58.73	Batch	PHB	[12]
*Bacillus subtilis*	Glucose	69.01	Batch	PHB	[3]
*Bacillus thuringiensis*	Glucose	11.30	Batch	PHB	[32]
*Bacillus thuringiensis* IAM12077	Glucose	64.16	–	PHB	[38]
*Lysinibacillus*sp. 3HHX	Glucose	80.94	Batch	PHB	[1]
*Paenibacillusdurus* BV-1	Fructose	0.93 g/l	–	PHB	[39]

### 2.2 Forms and taxonomy of biopolymers from *Bacillus*

The accumulated biopolymer PHAs comprises of 3-hydroxy fatty ester representing not only divergence but also complexity in their monomer classes. It is fascinating and the largest group of biopolyesters with more than 150 monomer compositions exhibiting diverse physical and chemical properties, and functionalities [43], [44]. Till now PHAs are grouped into three different categories based on the size, arrangements and number of carbon atom in the polymer, such as short chain length (scl-PHAs with C5 monomer), medium chain length (mcl-PHAs; with C6–C14 monomers) and long chain length (lcl-PHA; with ≥ C14 monomers) respectively [45].

Moreover, the homo and heteropolymers of PHAs corresponds to the presence of more than one type of hydroxyalkanoate monomers. The molecular weight of the polymer ranges from 2 × 10^5^ to 3 × 10^6^ Da, which is based on the type of microbial strain, upstream and downstream processing employed in the production method [46]. *Bacillus* species are also reported to accumulate heteropolymers of scl- to mcl-PHAs including P(3HB-co-3HV), P(3HB-co-3HHx) and P(3HB-*co*-4HB) with c-butyrolactone or e-caprolactone as C-source in the production media [47]. Though various PHA monomers are produced by *Bacillus* species *in vitro*, very few such as PHB, PHBV and PHBH have en routed for pilot-scale production [48].

### 2.3 Biochemical pathway of PHAs synthesis

Bacteria have the ability to synthesize PHAs in the stationary as well as exponential growth phases. Non-growth associated PHAs accumulation occurs in the stationary phase of bacterial growth with limitation of N, P, Mg and oxygen and excess carbon sources; however growth associated PHAs production takes place under balanced condition. Notably, most of the *Bacillus* species accumulate PHAs by adopting growth associated and non-growth associated mechanism [6], [49] as compared to other genera. Biosynthetic pathway of PHAs production varies among microbial groups. So far eight different pathways of microbial PHAs synthesis have been reported [13]. PHB, the most common homopolymer of PHAs synthesis starts from metabolism of glucose to generate acetyl-CoA and NADPH through the glycolytic and pentose phosphate pathways. Then, the two acetyl- CoA molecules condensed by *β*-ketothiolase (*PhaA*) into acetoacetyl-CoA and subsequently reduced to 3-hydroxybutyryl-CoA by acetoacetyl-CoA dehydrogenase (*PhaB*) using NADPH as a cofactor and finally polymerized into PHB by P(3HB) polymerase (*PhaC*) [6], [45], [50]. Thus, the NADPH is involved in reduction of acetoacetyl-CoA to 3-hydroxybutyryl-CoA due to over expression of the *zwf* and *gnd* genes that encode glucose 6-phosphate and 6-phosphogluconate dehydrogenase respectively [51]. As a matter of fact, the PHB production has been increased by raising the ratio of NADPH to NADP^+^.

**Fig. 1:**
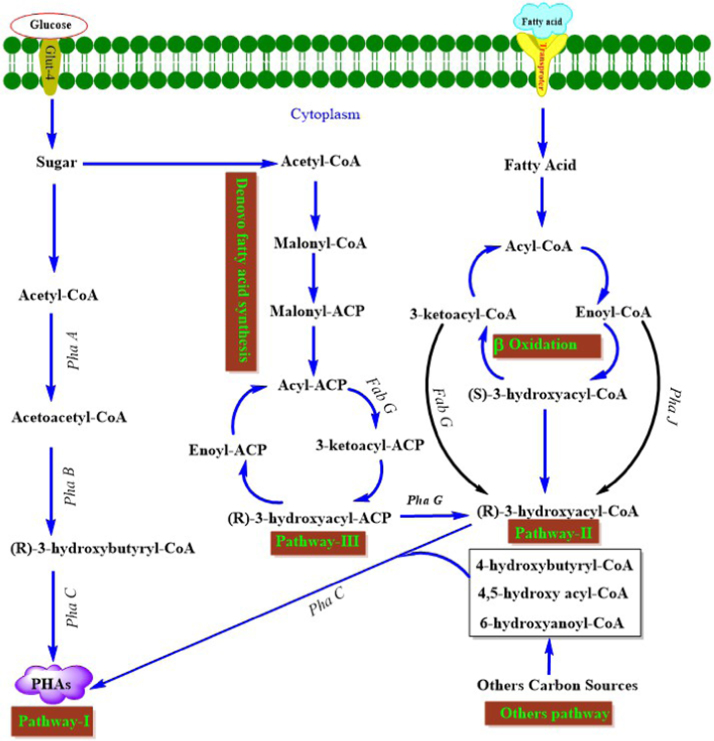
Metabolic pathways for synthesis of PHAs by bacteria [52].

**Fig. 2:**
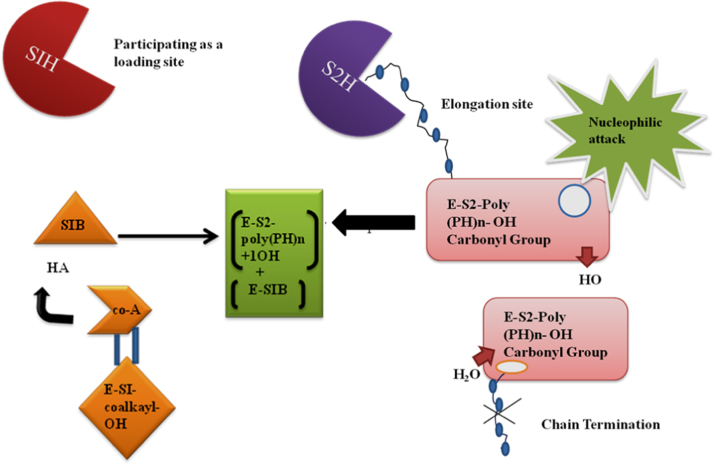
Molecular mechanism of activity of PHAs synthase gene.

Carbon sources in bacteria are metabolized differentially. So far three pathways for the synthesis of monomers of PHAs in bacteria have been well-studied (Fig. 1). Pathway I utilize sugars like glucose and fructose to yield PHB homopolymer. Copolymers are produced through pathway II and III [53], [54]. A contemporary hypothesis for the reaction mechanism of PHA synthases was proposed based on a model by Griebel et al. [55] with two thiolates taking part in the covalent catalysis of polyester synthesis [56]. PHAs synthesis takes place at the thiolate groups (SIH and S2H) of the PHA synthases (E), where one thiolate participates as loading site and the second thiolate acts as the elongation site. This mechanism suggested that one thiol group (SIB) receives a hydroxyalkanoic acid from Co-A thioester which is covalently bonded to the thiol group (E-S I-COAlkyl-OH) and the Co-A is released. However, the increasing polyester chain remains bonded to the second thiol group to form [E-S2-poly(PH)n-OH]. This complex, [E-S2-poly(PH)n-OH] then transfers the free hydroxyl group upon a nucleophilic attack of the hydroxyl oxygen atom on the carbonyl carbon atom giving rise to (E-S,-poly(HA)n^+^,-OH). A subsequent trans-esterification of the elongated polyester chain from S1 to S2 results in [E-S,-poly(HA)n+l-OH] + (E-S1B), and the latter can now accept the next hydroxyalkanoic acid from a Co-A thioester (Fig. 2). Moreover, *Bacillus* species [31], [57], [58], [59], [60] not only synthesize PHB by non-growth associated mechanism which is operated in nitrogen imitating condition but also by growth associated mechanism [61], [62], [63], [64], where high amount of nitrogen doesn’t affect the PHB production negatively [65], [66], reasonably it diverts the TCA cycle intermediates towards PHB biosynthesis [67].

### 2.4 Genes and operons of Bacillus involved in PHAs synthesis

The genes and enzymes regulating biogenesis of polyhydroxyalkanoates have distinct characteristics and depend on the bacterial strain. The ability of a bacterial isolate to synthesize a particular PHA is due to substrate specificity of the key enzyme PHA synthase. Extensive research to study PHA synthases present in the bacterial domain has been fervent,where these are categorized into four different classes. Moreover, the classification is not only based on substrate specificity of enzyme but also subunit composition [45]. These are PHA synthases class I that utilises CoA thio-esters of 3-HAs, 4-HAs and 5-HAs, and class II polymerases that have specificity towards CoA thio-esters of 3-HAs, 4-HAs and 5-HAs. Notably, these classes of enzymes are expressed by *phaC* gene. The Class III synthase enzyme is composed of two subunits such as *PhaE* and *PhaC*, with molecular weight 40 kDa and that have parallel substrate specificities to class I and have the potentiality to polymerise 3-HAs. However, Class IV synthases enzyme bear a resemblance to the class III PHA synthases, but the *PhaE* subunit is replaced by *PhaR* coded by *phaC* and *phaR* gene to synthesize polyhydroxyalkanoates [45].

**Fig. 3:**
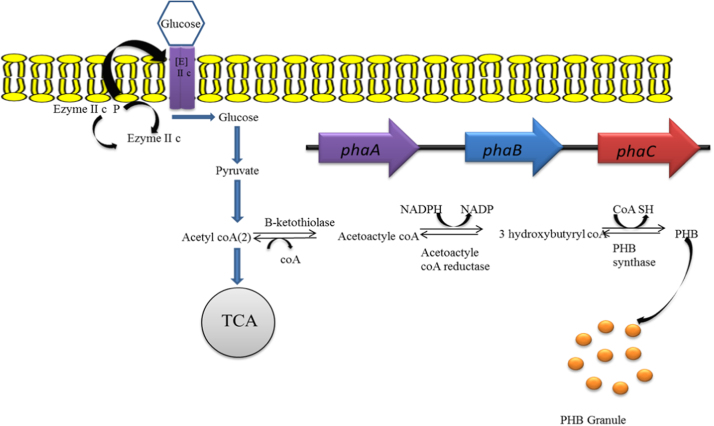
Generalized genetic mechanism of PHAs synthesis in bacteria.

Research findings [45] also revealed the presence of more than 59 genes associated with PHA synthesis from 45 distinguished bacterial species with varying nucleotide sequence. Though PHA synthase genes vary in number, they mainly occur in two or more different copies in different bacterial strains. These distinct types of the *phaC* gene present in some bacteria also regulate PHAs biosynthesis [68]. Certain PHAs producing bacteria possess a type I-PHA synthase gene clusters composed of the *β*-ketothiolase (*phaA*), acetoacetyl-CoA reductase (*phaB*), PHA synthase (*phaC*) and structural genes occurring in varying arrangements, as observed in *Pseudomonas*sp. 61-3, *R. eutropha*, *Acinetobacter* sp. RA3849, *A. latus* and *B. cepacia* [69], [70], [71], [72], [73] (Fig. 3). These three genes constitute an operon in certain bacterial strains, however other bacterial strains have some additional genes involved in PHAs metabolism are also present in the particular clusters [69], [74], [75]. Majority of PHAs synthesizing bacteria possess type I PHA synthase not positioned close to each other [71], [76]. On the other hand, few *Pseudomonas* species have two dissimilar PHA synthase genes clustered in the genome with the same point of reference and separated by a gene that encodes PHA depolymerize (*phaZ*)as revealed in *P. putida* strainU, *P. oleovorans, P. aeruginosa* and *P. mendocina* [77].

## 3 Production and characterization of *Bacillus* generated biopolymer

### 3.1 Use of suitable raw materials for biopolymer production

PHAs production has been observed by culturing *Bacillus* species in synthetic culture media [14], [24], [32], [35] since last more than three decades, but the media composition, biopolymer yield and the production cost vary greatly between reports. The raw material cost is predominantly an important factor affecting the overall economics of large-scale PHAs production [78]. Thus, the cost-effective mass production of PHAs production is inherently tied with the development of efficient submerged fermentation technology from low-cost carbon sources. Utilization of cheap raw materials as carbon sources concurrently reduces the cost of manufacture of value-added products [79]. Thus, several inexpensive carbon sources such as sugarcane molasses, beet molasses, date syrup, whey and activated sludge are most commonly used for PHAs production.

Moreover, a number of *Bacillus* species have been reported to produce PHAs from different low cost substrates or crude raw materials [11], [29], [30], [31]. The comparative PHB yields by *Bacillus* species in activated sludge to synthetic medium were 74% and 76.32% (DCW) [29], [35] as depicted in Table 1b. This validates the ability of *Bacillus* species to utilize diverse complex starch substrates and its dependence on type of the substrate and enzyme involved in fermentation process. It is pertinent to mention that, *Bacillus* species are well recognized for their capability to hydrolyze starch into simple sugars such as maltose & glucose by amylase and pullulanases enzyme, favoring growth as well as for PHAs production [80], [81]. PHAs productions employing starchy raw materials also require less amount of energy for liquefaction and saccharification of starch. Narayanan and Ramana (2012) [35] have reported an enhanced PHB production using *Bacillus mycoides* DFC1. Though, predominant Gram negative bacteria like *Cupriavidus necator* and *Alcaligens latus* have the potential to produce significant amount of PHB such as 80% and 88% DCW respectively, however, the level of endotoxin in the commercial PHAs can reach up to 120 U/g [82], [83]. Thus, endotoxin free PHAs production is highly indispensible using *Bacillus* species.

**Table 1b:** PHAs produced from low cost raw materials by different species of *Bacillus*.

***Bacillus*****sp.**	**Substrate**	**PHAs yields (% of DCW)**	**Fermentation**	**PHA type**	**Reference**
*Bacillucereus* PHA 008	Palm oil mill effluent	64.09	Batch	P(3HB)	[31]
*Bacillus megaterium*	Beet molasses, date syrup	50.00	Feed Batch	P(3HB)	[21]
*Bacillus megaterium* A9	Activated sludge	74.00	Feed Batch	PHB	[29]
*Bacillus megaterium* ATCC 6748	Sugarcane molasses	43.00	Batch	PHB	[23]
*Bacillumegaterium* BA-019	Molasses	42.10	Feed Batch	PHB	[25]
*Bacillumegaterium* OU303A	Glycerol	62.43	Batch	PHB	[24]
*Bacillumegaterium* P7	Yeast extract, Peptone	14.04	Batch	PHB	[22]
*Bacillus* sp.	Date syrup	70.50	–	PHAs	[11]
*Bacillus* sp. AS 3-2	Yeast extract	59.90	Batch	2-methyl-3-HB	[33]
*Bacillus subtilis*	Cashew fruits drink	4.40	Batch	PHB	[30]
*Bacillus subtilis*	Fish solid waste	70.00	Batch	PHB	[61]

### 3.2 Techniques involved in characterization of biopolymers

Although several methods used in extraction of PHAs content in bacteria have been described, many are time-consuming, procedurally tough, dependent on organic solvents, involve multiple purification steps and arduous dispersal approach of sodium hypochlorite, chloroform & digesting enzymes [84], [85]. These technologies are primarily cost and time intensive thus decreasing the efficacy of downstream processing as well as causing eco-pollution. Strazzullo et al. [86] proposed an efficient, downstream processing for PHAs extraction using sodium dodecyl sulphate with shaking to disperse microbial biomass in distilled water, heat treatment and washing. PHAs are structurally and thermally characterized by employing modern sophisticated methodologies [37] such as Fourier Transform Infrared Spectroscopy (FTIR), Nuclear Magnetic Resonance (NMR), Gas Chromatography Mass Spectroscopy (GCMS), High Performance Liquid Chromatography (HPLC), Liquid Chromatography Mass Spectroscopy (LCMS), X-Ray Diffraction (XRD), & X-ray Photoelectron Spectroscopy (XPS) and Gel Permeation Chromatography (GPC), Differential Scanning Colorimetry (DSC) and Thermo Gravimetric Analysis (TGA) respectively. In addition, the biodegradability and biocompatibility of the biopolymer (PHAs) are characterized by open windrow composting and Fluorescence Activated Cell Sorting (FACS) technology.

## 4 Properties and applications of *Bacillus* biopolymers

### 4.1 Significant properties of biopolymers

The PHAs from *Bacillus* species are closer than other genera to polypropylene in terms of thermal and other relevant properties [23], [24], [25], [37]. Bora et al. [87] reported a novel biopolymer by *Bacillus megaterium* strain with comparatively better properties like high melting stability, 44.09% crystalinity, 42 MPa tensile strength and 142% elongation-to-break with commercial polypropylene. Biopolymers by *Bacillus* are obviously biodegradable carried out by soil microbial communities which are influenced primarily by the polymer chemical composition, temperature, humidity and the active microbial consortia. PHAs degradation is enhanced by a decrease in the molecular weight of polymer and an increase in the degree of crystalinity. The number of potential PHAs degrader evolving at the surface of the polymer is lower than the number of associated bacteria. Some dominant soil PHA-degraders are the bacterial genera *Bacillus*, *Xanthomonas, Stenotrophomonas*, *Pseudomonas*, *Acinetobacter* & *Variovorax*, *Schlegelella*, *Azospirillum* and moulds like *Acremonium*, *Penicillium*, *Verticillium, Paecilomyces*, and *Zygosporium* [88], [89], [90].

### 4.2 Blending: an alternative approach for strengthening biopolymers

Various blends and composites to make the biopolymers suitable to market by enhancing their mechanical strength and reducing the cost have been tried since long. Their hydrophilic nature has helped in developing eco-friendly composites. Blends and multilayers of natural biopolymers with other polymers from sustainable resources can be targeted for their property improvisation. This process also helps to develop cost affordable biopolymer with significant performance. Most widely used natural polymer blends include starch, cellulose and rubber. Starch is the most accepted blending material because of its intrinsic biodegradability and renewability. Aliphatic polyesters are also recognized for their biodegradability and susceptibility for hydrolytic degradation. Such blending significantly increases the thermo-mechanical stability of PHB. Other advantage of blending is the cyto-compatibility to use these materials as biomaterials. Mechanical properties such as elevated Young's modulus and elongation to break the biopolymer matrix are the added advantages of the blended materials.

### 4.3 Commercial applications of biopolymer

Due to its biocompatibility and biodegradability PHAs has a wide range of potential applications such as in packaging, coating material, polymer films, non-woven materials, sutures and pharmaceutical products [91], [92], [93], [94], [95], [96], [97] to its negligible cytotoxicity, it is also being used in surgery, pharmacology, trans-plantology and tissue engineering [92], [98]. P(3HB-*co*-4HB) have been validated as scaffold in tissue engineering [83], P(3HB) as the pulmonary artery for the regeneration of arterial tissue [99], P(4HB) for preparing autologous cardiovascular tissue [100] and P(3HB-*co*-3HHx) in tissue engineering as well as for controlled drug-release [101], [102], [103] respectively. PHAs are also being used as cosmetic oil-blotting film [104], skincare products, potential source of organic acids supplement in animal feed and acts as an antimicrobial agent in animal production.

## 5 Prospects and challenge of using *Bacillus* for biopolymer production

**Fig. 4:**
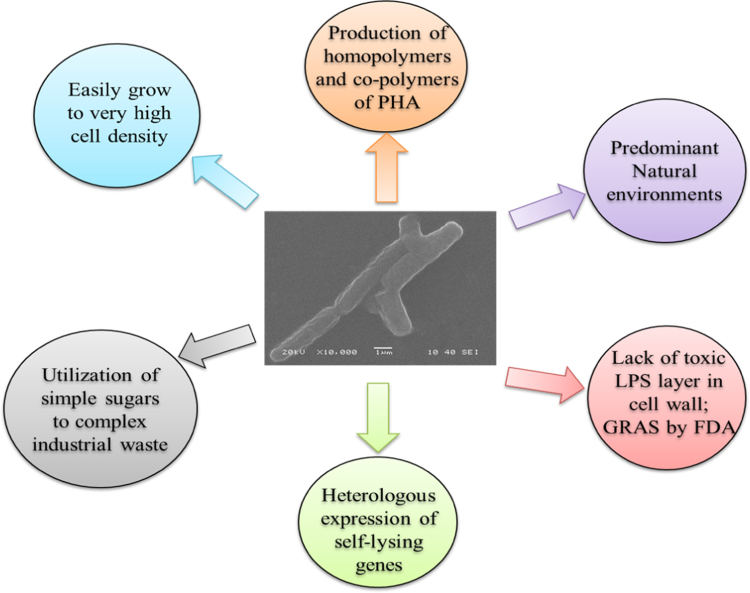
Advantages of using *Bacillus* species for large scale production of biopolymers.

As most *Bacillus* species are recognized as safe by the Food and Drug Administration (USFDA), it is an additional benefit for its biotechnological applications. Use of *Bacillus* species has been widely appreciated owing to their many other properties like production of extracellular metabolites, bioremediation and bioenergy production. *Bacillus* species reportedly produce 11–69% higher amount of PHAs compared to other bacterial strains [28], the most potent ones being *B. amyloliquefaciens*, *B. laterosporus*, *B. mycoides*, *B. licheniformis*, *B. circulans, B. macerans*, *B. cereus*, *B. firmus*, *B. subtilis*, *B. coagulans*, *B. sphaericus*, *B. brevis, B. megaterium*, and *B. thuringiensis*. Another advantage of *Bacillus* species as PHAs producer is its heterogeneous representation. As *B. subtilis* is the first Gram-positive to be sequenced completely, it has opened a plethora of functional analysis of Gram-positive bacteria. *Bacillus* species reportedly produces PHAs homo-polymers and co-polymers that increase the diverse nature of the synthesized PHAs [14], [68]. They are easily grown utilizing simple sugars to complex industrial wastes. Predominance in the nature and lack of the lipopolysaccharide layer are the added advantages for the use of *Bacillus* species in industrial scale preparation of biopolymers (Fig. 4).

## 6 Future perspectives

Bacterial biopolymer production and its numerous alternate applications have pushed the bio-industrial sector for its possible commercial-scale production. Wide use of bioplastics can potentially address many potential environmental hazards overcoming the dependence on petroleum to produce plastics, and reduction in CO_2_ emission thereby protecting the environment. Bioplastics are being used as biofuels that has bred huge attentions among the researchers to explore this field. A major problem regarding the use of bioplastics, however, is its high cost. Though many works have been carried out to decrease their production cost, still miles to go to achieve a gold standard in this regard. High value-added applications could be of an immediate interest explored them in surgical and therapeutic applications. Another potential application could be the use of their surface-binding proteins like *PhaP*, *PhaZ* and *PhaC* as drug delivery tools, a possible application in nano-medicine. Genetic modification of bacterial strains to maximize production of biopolymers can be a future research target.

## 7 Conclusion

In view of the recent advances in biopolymer research, primarily the PHAs have significant impact as a potential substitute of petro-chemical based plastics. The major challenge for the economical production of biopolymers (PHAs) depends on the selection of potential microbes by polyphasic approach and a cost-effective production approach. This suggests selection of suitable *Bacillus* species capable of efficient consumption and bioconversion of inexpensive substrates into a broad range of PHAs with diverse properties and applications. Among the various explored waste material, activated sludge seems to be the most promising for the *Bacillus* species. Combining the batch and fed-batch fermentations for enhanced productivity compared to the other methods available in the public domain can be another process intervention. Considering the controllable nature of chemostat, fed-batch fermentation seems to great potential to enhance productivities. All such efforts at the laboratory scale will need to be validated at pilot-scale for future industrial production and wide application of this biopolymer to tap the application potential of such bacterial species in general, and the genus *Bacillus* in particular.

## References

[1] Mohapatra S., Samantaray D.P., Samantaray S.M., Mishra B.B., Das S., Majumdar S., Pradhan S.K., Rath S.N., Rath C.C., Akthar J., Achary K.G. (2016). Structural and thermal characterization of PHAs produced by *Lysinibacillus* sp. through submerged fermentation process. Int. J. Biol. Macromol..

[2] Chanprateep S. (2010). Current trends in biodegradable polyhydroxyalkanoates. J. Biosci. Bioeng..

[3] Mohapatra S., Mohanta P.R., Sarkar B., Daware A., Kumar C., Samantaray D.P. (2015). Production of polyhydroxyalkanoates (PHAs) by *Bacillus* strain isolated from waste water and its biochemical characterization. Proc. Natl. Acad. Sci. USA.

[4] Koller M., Gasser I., Schmid F., Berg G. (2011). Linking ecology with economy: insights into polyhydroxyalkanoates producing microorganisms. Eng. Life Sci..

[5] Page W.J. (1995). Bacterial polyhydroxyalkanoates, natural biodegradable plastics with a great future. Canad. J. Microbiol..

[6] Lee S.Y. (1996). Plastic bacteria? Progress and prospects for polyhydroxyalkanoates production in bacteria. Trends Biotechnol..

[7] Poli A., Paola D.D., Abbamondi G.R., Nicolaus B. (2011). Synthesis, production and biotechnological applications of exopolysaccharides and polyhydroxyalkanoates by Archaea. Archaea.

[8] Lemoigne M. (1926). Produits de deshydrationet the polymerization de l′acide b-oxybutirique. Bull. Soc. Chim. Biol..

[9] Madison L.L., Huisman G.W. (1999). Metabolic engineering of poly (3-hydroxyalkanoates): from DNA to plastic. Microbiol. Mol. Biol. Rev..

[10] Biedendieck R., Gamer M., Jaensch L., Meyer S., Rohde M., Deckwer W.D., Jahn D.A. (2007). Sucrose inducible promoter system for the intra- and extracellular protein production in *B. megaterium*. J. Biotechnol..

[11] Khiyami M.A., Fadual S.M., Bahklia A.H. (2011). Polyhydroxyalkanoates production via *Bacillus* plastic composite support (PCS) biofilm and date palm syrup. J. Med. Plants Res..

[12] Israni N., Shivakumar S. (2013). Combinatorial screening of hydrolytic enzymes and PHAs producing *Bacillus* sp. for cost effective production of PHAs. Int. J. Pharma Biosci..

[13] Chen G.Q. (2010). Plastics from bacteria: natural functions and applications. Microbiol. Monogr..

[14] Valappil S.P., Rai R., Bucke C., Roy I. (2007). Polyhydroxyalkanoate biosynthesis in *Bacillus cereus* SPV under varied limiting conditions and an insight into the biosynthetic genes involved. J. Appl. Microbiol..

[15] Wakisaka Y., Masaki E., Nishimoto Y. (1982). Formation of crystalline δ-endotoxin or poly β-hyroxybutyrate acid granules by as porogenous mutants of *Bacillus thuringiensis*. Appl. Environ. Microbiol..

[16] Lee S.Y., Chang H.N. (1995). Production of poly- hydroxyalkanoic acid. Adv. Biochem. Eng. Biotechnol..

[17] Cavalheiro J.M.B.T., Almeida M.C.M.D., Grandfils C., Fonseca M.M.R. (2009). Poly (3-hydroxybutyrate) production by *Cupriavidus necator* using waste glycerol. Process Biochem..

[18] Parveez G.K.A., Bohari B., Ayub N.H., Yunus A.M.M., Rasid O.A., Hashim A.T., Ishak Z., Manf M.A.A., Din A.K., York G., Jo Y.B., Sinskey A.J. (2008). Transformation of PHB and PHBV genes driven by maize ubiquitin promoter into oil palm for the production of biodegradable plastics. J. Oil. Palm. Res..

[19] Rehm B.H.A. (2007). Biogenesis of microbial polyhydroxyalkanoates granules: a platform technology for the production of tailormade bio-particles. Curr. Issu. Mol. Biol..

[20] Nicolaus B., Lama L., Esposito E. (1999). *Haloarcula* spp. able to biosynthesize exo- and endo-polymers. J. Indust. Microbiol. Biotechnol..

[21] Omar S., Rayes A., Eqaab A., Steinbuchel A. (2001). Optimization of cell growth and poly (3-hydroxybutyrate) accumulation on date syrup by a *Bacillus megaterium* strain. Biotechnol. Lett..

[22] Yilmaz H., Soran H., Beyatli Y. (2005). Determination of poly-β-hydroxybutyrate (PHB) production by some *Bacillus* spp. World J. Microbiol. Biotechnol..

[23] Chaijamrus S., Udpuay N. (2008). Production and characterization of polyhydroxybutyrate from molasses and corn steep liquor produced by *Bacillus megaterium* ATCC 6748. Agricul. Eng. Int. CIGRE J. Manuscript.

[24] Reddy S.V., Thirumala M., Mahmood S.K. (2009). Production of PHB and P (3HB-co-3HV) biopolymers by *Bacillus megaterium* strain OU303A isolated from municipal sewage sludge. World J. Microbiol. Biotechnol..

[25] Kulpreecha S., Boonruangthavorn A., Meksiriporn B., Thongchul N. (2009). Inexpensive fed-batch cultivation for high poly (3-hydroxybutyrate) production by a new isolate of *Bacillus megaterium*. J. Biosci. Bioeng..

[26] Joshi P.A., Jaysawal S.R. (2010). Isolation and characterization of poly-β-hydroxyalkanoate producing bacteria from sewage sample. J. Cell Tissue Res..

[27] Singh G., Mittal A., Kumari A., Goel V., Aggarwal N.K., Yadav A. (2011). Optimization of poly-B-hydroxybutyrate production from *Bacillus* species. Eur. J. Biol. Sci..

[28] Aarthi N., Ramana K. (2011). Identification and characterization of polyhydroxybutyrate producing *Bacillus cereus* and *Bacillus mycoides* strains. Int. J. Environ. Sci..

[29] Chookietawattana K., Khonsarn N. (2011). Biotechnological conversion of wastewater to polydydroxyalkanoates by *Bacillus* in a sequencing batch reactor. World Appl. Sci. J..

[30] Ghate B., Pandit P., Kulkarni C., Mungi D.D., Patel T.S. (2011). PHB production using novel agro-industrial sources from different *Bacillus* species. Int. J. Pharma Biosci..

[31] Sangkharak K., Prasertsan P. (2012). Screening and identification of polyhydroxyalkanoates producing bacteria and biochemical characterization of their possible application. J. Gen. Appl. Microbiol..

[32] Patel S.K.S., Singh M., Kalia V.C. (2011). Hydrogen and polyhydroxybutyrate producing abilities of *Bacillus* spp. from glucose in two stage system. Ind. J. Microbiol..

[33] Shah K.R. (2012). FTIR analysis of polyhydroxyalkanoates by novel *Bacillus* sp. AS 3-2 from soil of Kadi region, North Gujarat, India. J. Biochem. Technol..

[34] Abinaya R.V., Balasubramanian V., Ramesh N., Natrajan K., Rajeshkannan V. (2012). Exploration of polyhydroxyalkanoates production from rhizosphere soil bacteria. Envis Newslett..

[35] Narayanan Ramana K.V. (2012). Polyhydroxybutyrate production in *Bacillus mycoides* DFC1 using response surface optimization for physico-chemical process parameters. 3 Biotechnology.

[36] Berekaa M.M., Thawadi A.M.A. (2012). Biosynthesis of polyhydroxybutyrate (PHB) biopolymer by *Bacillus megaterium* SW1-2: application of Box-Behnken design for optimization of process parameters. Afr. J. Microbiol. Res..

[37] Contreras A.R., Koller M., Dias M.M.D., Calafell-Monfort M., Braunegg G., Marques-Calvo M.S. (2013). High production of poly (3-hydroxybutyrate) from a wild *Bacillus megaterium* Bolivian strain. J. Appl. Microbiol..

[38] Gowda V., Shivakumar S. (2013). Poly (3)hydroxybutyrate (PHB) production in *Bacillus thuringiensis* IAM 12077 under varied nutrient limiting conditions and molecular detection of class IV pha synthase gene by PCR. Int. J. Pharm. Biol. Sci..

[39] Hungund B., Shyama V.S., Patwardhan P., Saleh A.M. (2013). Production of polyhydroxyalkanoate from *Paenibacillus durus* BV-1 isolated from oil mill soil. J. Microb. Biochem. Technol..

[40] Tanamool V., Imai T., Danvirutai P., Kaewkannetra P. (2013). Biopolymer generation from sweet sorghum juice: screening, isolation, identification and fermentative polyhydroxyalkanoate production by *Bacillus aryabhattai*. Turk. J. Biol..

[41] Raj A., Ibrahim V., Devi M., Sekar K.V., Yogesh B.J., Bharathi S. (2014). Screening, optimization and characterization of polyhydroxyalkanoates(PHA) produced from microbial isolates. Int. J. Curr. Microbiol. Appl. Sci..

[42] Dash S., Mohapatra S., Samantaray D.P., Sethi A.K. (2014). Production of polyhydroxyalkanoates by sugar cane rhizospheric soil bacterial isolates. J. Pur. Appl. Microbiol..

[43] Steinbüchel A., Valentin H.E. (1995). Diversity of bacterial polyhydroxyalkanoic acids. FEMS Microbiol. Lett..

[44] Hazer B., Steinbüchel A. (2007). Increased diversification of polyhydroxyalkanoates by modification reactions for industrial and medical applications. Appl. Microbiol. Biotechnol..

[45] Rehm B.H.A. (2003). Polyester synthases: natural catalysts for plastics. Biochem. J..

[46] Keshavarz T., Roy I. (2010). Polyhydroxyalkanoates: bioplastics with a green agenda. Curr. Opin. Microbiol..

[47] Labuzeck S., Radecka I. (2001). Biosynthesis of terco-polymer by *Bacillus cereus* UW85. J. Appl. Microbiol..

[48] Avella M., Bonadies E., Martuscelli E. (2001). European current standardization for plastic packaging recoverable through composting and biodegradation. Polym. Test..

[49] Borah B., Thakur P.S., Nigam J.N. (2002). The influence of nutritional and environmental conditions on the accumulation of poly-beta-hydroxybutyrate in *Bacillus mycoides* RLJ B-017. J. Appl. Microbiol..

[50] Kalia V.C., Lal S., Cheema S. (2007). Insight in to the phylogeny of polyhydroxyalkanoates biosynthesis: horizontal gene transfer. Gene.

[51] Lim S.J., Jung Y.M., Shin H.D., Lee Y.H. (2002). Amplification of the NADPH-related genes zwf and gnd for the oddball biosynthesis of PHB in an *E. coli* transformant harbouring a cloned phb CAB operon. J. Biosci. Bioeng..

[52] Verlinden R.A.J., Hill D.J., Kenward M.A., Williams C.D., Radecka I. (2007). Bacterial synthesis of biodegradable polyhydroxyalkanoates. J. Appl. Microbiol..

[53] Aldor I.S., Keasling J.D. (2003). Process design for microbial plastic factories: metabolic engineering of polyhydroxyalkanoates. Curr. Opin. Biotechnol..

[54] Steinbüchel A., Lütke-Eversloh T. (2003). Metabolic engineering and pathway construction for biotechnological production of relevant polyhydroxyalkanoates in microorganisms. Biochem. Eng. J..

[55] Griebel R., Smith Z., Merrick J.M. (1968). Metabolism of poly-beta-hydroxybutyrate. I. Purification, composition, and properties of native poly-beta-hydroxybutyrate granules from *Bacillus megaterium*. Biochemistry.

[56] Anderson A.J., Dawes E.A. (1990). Occurrence, metabolism, metabolic role, and industrial uses of bacterial polyhydroxyalkanoates. Microbiol. Rev..

[57] Mohapatra S., Samantaray D.P., Samantaray S.M. (2014). Phylogenetic heterogeneity of the rhizospheric soil bacterial isolates producing PHAs revealed by comparative analysis of 16s-rRNA. Int. J. Curr. Microbiol. Appl. Sci..

[58] Faccin D.J.L., Rech R., Secchi A.R., Cardozo N.S.M., Ayu M.A.Z. (2013). Influence of oxygen transfer rate on the accumulation of poly(3-hydroxybutyrate) by *Bacillus megaterium*. Process Biochem..

[59] Chaudhry W., Jamil N., Ali I., Ayaz M., Hasnain S. (2011). Screening for polyhydroxyalkanoate (PHA) producing bacterial strains and comparison of PHAs production from various inexpensive carbon sources. Ann. Microbiol..

[60] Khanna S., Srivastava A.K. (2005). Recent advances in microbial polyhydroxyalkanoates. Process Biochem..

[61] Mohapatra S., Sarkar B., Samantaray D.P., Daware A., Maity S., Pattanaik S., Bhattacharjee S. (2017). Bioconversion of fish solid waste into PHB using *Bacillus subtilis* based submerged fermentation process. Environ. Technol.

[62] Kumar P., Ray S., Patel S.K.S., Lee J.K., Kalia V.C. (2015). Bioconversion of crude glycerol to polyhydroxyalkanoate by *Bacillus thuringiensis* under non-limiting nitrogen conditions. Int. J. Biol. Macromol..

[63] Saraphirom P., Reungsang A., Plangklang P. (2013). Polyhydroxyalkanoates production from effluent of hydrogen fermentation process by *Cupriavidus* sp. KKU38. Environ. Technol..

[64] Lau N.S., Tsuge T., Sudesh K. (2011). Formation of new polyhydroxyalkanoate containing 3-hydroxy-4-methylvalerate monomer in *Burkholderia* species. Appl. Microbiol. Biotechnol..

[65] Mozumder M.S.I., Gonzalez L.G., Wever H.D., Volcke E.I.P. (2015). Poly (3-hydroxybutyrate) (PHB) production from CO_2_: model development and process optimization. Biochem. Eng. J..

[66] Kumar P., Patel S.K., Lee J.K., Kalia V.C. (2013). Extending the limits of *Bacillus* for novel biotechnological application. Biotechnol. Adv..

[67] Kumar T., Singh M., Purohit H.J., Kalia V.C. (2009). Potential of *Bacillus* sp. to produce polyhydroxybutyrate from biowaste. J. Appl. Microbiol..

[68] Steinbuchel A., Hein S. (2001). Biochemical and molecular basis of microbial synthesis of polyhydroxyalkanoates in microorganisms. Adv. Biochem. Eng. Biotechnol..

[69] Matsusaki H., Manji S., Taguchi K., Kato M., Fukui T., Doi Y. (1998). Cloning and molecular analysis of the poly (3-hydroxybutyrate) and poly (3-hydroxybutyrate-co-3-hydroxyalkanoate) biosynthesis genes in *Pseudomonas* sp. strain 61-3. J. Bacteriol..

[70] Valentin H.E., Steinbüchel A. (1993). Cloning and characterization of the *Methylobacteriumextorquens*polyhydroxyalkanoic-acid-synthase structural gene. Appl. Microbiol. Biotechnol..

[71] Valentin H.E., Steinbuchel A. (1994). Application of enzymatically synthesized short- chain- length hydroxy fatty acid co A thioester for assy of polyhydroxyalkanoic acid synthase. Appl. Microbiol. Biotechnol..

[72] Choi J., Lee S.Y. (1999). Factors affecting the economics of polyhydroxyalkanoate production by bacterial fermentation. Appl. Microbiol. Biotechnol..

[73] Rodrigues M.F.A., Vicente E.J., Steinbüchel A. (2000). Studies on polyhydroxyalkanoate (PHA) accumulation in a PHA synthase I-negative mutant of *Burkholderia cepacia* generated by homogenotization. FEMS Microbiol. Lett..

[74] Rehm B.H.A., Steinbüchel A. (1999). Biochemical and genetic analysis of PHA synthases and other proteins required for PHA synthesis. Int. J. Bio. Macromol..

[75] Satoh Y., Minamoto N., Tajima K., Munekata M. (2002). polyhydroxyalkanoate synthase from *Bacillus* sp. INTO05 is composed of *PhaC* and *PhaR*. J. Biosci. Bioeng..

[76] Qi Q., Rehm B.H.A. (2001). Polyhydroxybutyrate biosynthesis in *Caulobacter crescentus*: molecular characterization of the polyhydroxybutyrate synthase. Microbiology.

[77] García B., Olivera E.R., Minambres B., Fernández-Valverde M., Cañedo L.M., Prieto M.A., García J.L., Martínez M., Luengo J.M. (1999). Novel biodegradable aromatic plastics from a bacterial source, genetic and biochemical studies on a route of the phenylacetyl-CoA catabolon. J. Biol. Chem..

[78] Castilho L.R., Mitchell D.A., Freire D.M.G. (2009). Production of polyhydroxyalkanoates (PHAs) from waste materials and by-products by submerged and solid-state fermentation. Bioresour. Technol..

[79] Du C., Sabirova J., Soetaert W., Ki C.L.S. (2012). Polyhydroxyalkanoates production from low-cost sustainable raw materials. Curr. Chem. Biol..

[80] Schulein M., Pederson B.H. (1984). Characterization of new class of thermophillic pullulanases from *Bacillus acidopullulyticus*. Ann. N.Y. Acad. Sci..

[81] Atkins D.P., Kennedy J.F. (1985). The influence of and α-amylase upon the oligosaccharide product spectra of wheat starch hydrolysates. Starch.

[82] Lee S.Y., Choi K., Song J.Y. (1999). Removal of endotoxin during purification of poly (3-hydroxybutyrate) from Gram negative bacteria. Appl. Environ. Microbiol..

[83] Williams S.F., Martin D.P., Horowitz D.M., Peoples O.P. (1999). PHA applications: addressing the price performance issue: I. Tissue engineering. Int. J. Biol. Macromol..

[84] Braunneg G., Sonnleitner B., Lafferty R.M. (1978). A rapid gas chromatography method for determination of polyhydroxyalkanoates from microbial biomass. Appl. Microbiol. Biotechnol..

[85] Hahn S.Y., Chang Y.K., Kim B.S., Chang H.N. (1994). Communication to the editor optimization of microbial poly (3-hydroxybutyrate) recovery using dispersions of sodium hypochlorite solution and chloroform. Biotechnol. Bioeng..

[86] Strazzullo G., Gambacorta A., Vella F.M. (2008). Chemical-physical characterization of polyhydroxyalkanoates recovered by means of a simplified method from cultures of *Halomonas campaniensis*. World J. Microbiol. Biotechnol..

[87] Bora L., Das R., Gohain D. (2014). A novel melt stable and high tensile strength biopolymer (polyhydroxyalkanoates) from *Bacillus megaterium* (MTCC10086) and its characterization. J. Basic Microbiol..

[88] Boyandin A.N., Prudnikova S.V., Filipenko M.L., Khrapov E.A., Vasil’ev A.D., Volova T.G. (2012). Biodegradation of polyhydroxyalkanoates by soil microbial communities of different structures and detection of PHA degrading microorganisms. Appl. Biochem. Microbiol..

[89] Elbanna K., Lütke-Eversloh T., Jendrossek D., Luftmann H., Steinbüchel A. (2004). Studies on the biodegradability of polythioester copolymers and homopolymers by polyhydroxyalkanoate (PHA) degrading bacteria and PHA depolymerases. Arch. Microbiol..

[90] Kadouri D., Jurkevitch E., Okon Y. (2003). Poly β-hydroxybutyratedepolymerase (*PhaZ*) in *Azospirillumbrasilense*and characterization of a *phaZ* mutant. Arch. Microbiol..

[91] Otari S.V., Ghosh S.J. (2009). Production and characterization of the polymer polyhydroxy butyrate-co-polyhydroxy valerate by *Bacillus megaterium* NCIM 2475. Cur. Res. J. Biol. Sci..

[92] Porwal S., Kumar T., Lal S., Rani A., Kumar S., Cheema S., Purohit H.J., Sharma R., Patel S.K.S., Kalia V.C. (2008). Hydrogen and polyhydroxybutyrate producing abilities of microbes from diverse habitats by dark fermentative process. Bioresour. Technol..

[93] Patel S.K.S., Kumar P., Singh M., Lee J.K., Kalia V.C. (2015). Integrative approach to produce hydrogen and polyhydroxybutyrate from biowaste using defined bacterial cultures. Bioresour. Technol..

[94] Patel S.K.S., Lee J.K., Kalia V.C. (2016). Integrative approach for producing hydrogen and polyhydroxyalkanoates from mixed waste of biological origin. Int. J. Microbiol..

[95] Singh M., Kumar P., Patel S.K.S., Kalia V.C. (2013). Production of polyhydroxyalkanoate co-polymer by *Bacillus thuringiensis*. Int. J. Microbiol..

[96] Patel S.K.S., Singh M., Kumar P., Purohit H.J., Kalia V.C. (2012). Exploitation of defined microbial biodiversity for producing hydrogen and polyhydroxybutyrate from pea-shells. Biol. Bioeng..

[97] Noda. Process for recovering polyhydroxyalkanoates using air classification, U.S. Pat.5, 1998, pp. 849–854.

[98] Kumar P., Singh M., Mehariya S., Patel S.K.S., Lee J.K., Kalia V.C. (2014). Ecobiotechnological approach for exploiting the abilities of *Bacillus* to produce co-polymer of polyhydroxyalkanoate. Int. J. Microbiol..

[99] Shinoka T., Shum-Tim D., Ma P.X., Tanel R.E., Isogai N., Langer R., Vacanti J.P., Mayer J.E. (1998). Creation of viable pulmonary artery auto-grafts through tissue engineering. J. Thorac. Cardiovasc. Surg..

[100] Stock U.A., Sakamoto T., Hatsuoka S., Martin D.P., Nagashima M., Moran A.M., Moses M.A., Khalil P.N., Schoen F.J., Vacanti J.P., Mayer J.E. (2000). Patch augmentation of the pulmonary artery with bio-absorbable polymers and auto-logous cell seeding. J. Thorac. Cardiovasc. Surg..

[101] Shangguan Y.Y., Wang Y.W., Wu Q., Chen G.Q. (2006). The mechanical properties and *in vitro* biodegradation and biocompatibility of UV-treated poly (3-hydroxybutyrate-*co*-3-hydroxyhexanoate). Biomaterials.

[102] Qu X.H., Wu Q., Zhang K.Y., Chen G.Q. (2006). In vivo synthesis of poly (3-hydroxybutyrate-*co*-3-hydroxyhexanoate) based polymers: biodegradation and tissue reactions. Biomaterials.

[103] Singh M., Patel S.K.S., Kalia V.C. (2009). *Bacillus subtilis* as potential producer for polyhydroxyalkanoates. Microb. Cell Fact..

[104] Sudesh K., Loo C.Y., Goh L.K., Iwata T., Maeda M. (2007). The oil-absorbing property of polyhydroxyalkanoates films and its practical application: a refreshing new outlook for an old degrading material. Macromol. Biosci..

